# The Association Between Internet Searches and Moisturizer Prescription in Japan: Retrospective Observational Study

**DOI:** 10.2196/13212

**Published:** 2019-10-08

**Authors:** Wataru Mimura, Manabu Akazawa

**Affiliations:** 1 Department of Public Health and Epidemiology Meiji Pharmaceutical University Tokyo Japan

**Keywords:** internet, moisturizer, heparinoid, Google Trends, time series analysis, infodemiology

## Abstract

**Background:**

Heparinoid is a medication prescribed in Japan for skin diseases, such as atopic dermatitis and dry skin. Heparinoid prescription has increased with instances of internet blogs recommending its use as a cosmetic.

**Objective:**

This study aimed to examine the prescription trends in moisturizer use and analyze their association with internet searches.

**Methods:**

We used a claims database to identify pharmacy claims of heparinoid-only prescriptions in Japan. Additionally, we used Google Trends to obtain internet search data for the period between October 1, 2007, and September 31, 2017. To analyze the association between heparinoid prescriptions and internet searches, we performed an autoregressive integrated moving average approach for each time series.

**Results:**

We identified 155,733 patients who had been prescribed heparinoid. The number of prescriptions increased from 2011 onward, and related internet searches increased from 2012 onward. Internet searches were significantly correlated with total heparinoid prescription (correlation coefficient=.25, *P*=.005). In addition, internet searches were significantly correlated with heparinoid prescription in those aged 20-59 years at –1-month lag in Google Trends (correlation coefficient=.30, *P*=.001).

**Conclusions:**

Google searches related to heparinoid prescriptions showed a seasonal pattern and increased gradually over the preceding several years. Google searches were positively correlated with prescription trends. In addition, in a particular age group (20-59 years), prescriptions increased with the increase in internet searches. These results suggest that people obtained health-related information on the internet and that this affected their behavior and prescription requests.

## Introduction

Heparinoid is a transdermal medicine (ie, cream, ointment, lotion, spray, or gel) used as an anti-inflammatory aid, blood circulation promoter, and moisturizer in Japan [1]. It is generally prescribed to patients with thrombophlebitis, pain, and inflammatory disease caused by vascular insufficiency, chilblains, hypertrophic and keloid scars, keratodermia tylodes palmaris progressive, asteatosis, and posttraumatic swelling and pain [2]. Japanese guidelines for atopic dermatitis state that hydrophilic and water-absorptive ointment, including urea, heparinoid, and water-soluble collagen, should be used for dry skin [3]. Consequently, approximately 70% of patients younger than 15 years of age are prescribed moisturizers or protective agents [4], but the long-term trends in moisturizer prescription in Japan remain unknown.

In October 2017, the increase in heparinoid prescriptions became news in Japan. This was because the National Federation of Health Insurance Societies proposed that heparinoid should be eliminated from insurance if it was prescribed only for patients with dry skin [5]. The increase in prescriptions is considered to have occurred because blogs or social media recommended using moisturizer as a cosmetic. However, whether this is attributed to the dissemination of information via the internet remains unclear.

The internet is an important tool in searching for information. In Japan, 83.5% of people had used the internet during 2016 [6]. In particular, the proportion of usage in people aged 20-59 years has been more than 90% during the past several years. In addition, many people worldwide use the internet to seek health-related information on diseases and medicines [7-11]. They seek such information to understand their current health status, their disease, someone else’s disease, or prescription drugs, and so they are able to communicate with their physician [11-13]. This leads to an increase in their knowledge, satisfaction, and confidence as well as a reduction in their anxiety and stress. According to a previous study from 2007, although television (60.1%) and newspapers (50.3%) were major sources of health-related information, personal computers (23.8%) and mobile phones (6.0%) were also important tools for seeking such information via the internet [14]. We can easily guess that even more people are using the internet now given the further development of the internet since 2007.

Searching for health-related information on the internet has been shown to have positive effects on the frequency of visits to health professionals for those who seek such information compared to those who did not [15]. Health-related information seeking affects individuals’ demands for health care services and increases health care utilization. However, many people can obtain and disseminate information via the internet, regardless of whether health-related information is correct [16,17]. This field of study, called infodemiology, identifies the distribution and pattern of information and assesses subsequent changes in knowledge and health behavior. Sometimes, health-seeking behavior on the internet happens before changes occur in the actual health behavior [17,18]; we can obtain data about such health-seeking behavior from various sources, such as internet search engines, blogs, or social media [19]. Google facilitates the analysis of search queries using Google Trends [20], one of the most frequently used tools to analyze such data; it has been used to analyze the association between search queries and various topics. For instance, research examining the following topics has been conducted using internet search results: the prediction of influenza and infection [21,22], the seasonal change in depression [23], the influence of certain topics on suicide rate [24,25], the relationship between the search volume of certain topics and cancer incidence [26], the impact of celebrities’ diseases on public opinions [27], and the monitoring of drug utilization [28].

Although many studies have used Google Trends, none have examined the relationship between internet searches and prescriptions in Japan. Therefore, the purpose of this study was to examine the trend in prescription moisturizer use and to analyze its association with internet searches.

## Methods

### Claims Database

We used the administrative claims database provided by JMDC Inc [29], which is one of the largest commercial databases in Japan [30]. Since 2005, it has held anonymized data for a population of 5,600,000, including individuals 65 years of age and younger as well as employees of large corporations and their dependents. The database consists of inpatient data, outpatient data, pharmacy data, diagnosis procedure combination data, and insured-population data. In addition, it contains anonymous data regarding patient characteristics; diagnostic codes according to the International Statistical Classification of Diseases and Related Health Problems, Tenth Revision (ICD-10); surgery; procedures; drug prescriptions; medical institutions; costs; and prescription dates. Regarding drug data, it contains the World Health Organization Anatomical Therapeutic Chemical codes, generic drug names, product names, prescription dates, and dispensing dates. The database also includes data regarding insured people; therefore, we were able to not only obtain information about populations, but also determine if patients had changed medical institutions from hospitals to clinics.

We used pharmacy data for the period from October 2007 to September 2017, because television and newspaper coverage of moisturizer prescription increased in October 2017. Therefore, to assess the association between prescription moisturizer and internet searches, we decided to use the data from October 2007 until September 2017. Additionally, we identified heparinoid prescription—the World Health Organization Anatomical Therapeutic Chemical code: C05B01. We focused on heparinoid prescriptions only, as prescriptions of heparinoid with other drugs, such as antihistamines, might not have been affected by internet searches. We defined prescriptions from insurance identification numbers, prescription dates, and medical facilities. The study protocol was approved the ethics committee at Meiji Pharmaceutical University (approval number: 2959).

### Internet Search Data

The internet search data was obtained from Google Trends, which is a product provided by Google. We included geography and time range in the Google Trends search terms. Search trend data were available for the period from 2004 to 36 hours before our search on April 23, 2018. Search results were shown on a scale ranging from 0 to 100, based on the ratio of searches on a given topic to searches on all topics. For example, imagine we were obtaining search results of the word “A” for every month for a year. If “A” was searched most frequently in April, the search results have a score of 100 for April, and the search frequency of other months are scored relative to the frequency in April. When we compare two words, “A” and “B,” the most frequently searched month is identified for both “A” and “B” [26].

We chose three search terms that we considered most closely related to heparinoid. We used the Japanese “
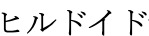
” (Hirudoid) and “
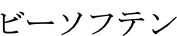
” (Besoften), which are the names of major products containing heparinoid in Japan. In addition, we entered “
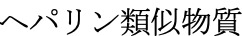
” (heparinoid), which is the generic drug name. We performed the search on April 23, 2018, and compared the three terms in Google Trends from October 1, 2007, to September 31, 2017, in Japan.

### Statistical Analysis

We performed a descriptive analysis to examine patient characteristics, monthly internet search data, and prescription trends. Patient age was stratified into three groups: 0-19 years, 20-59 years, and ≥60 years. This is because previous reports showed that moisturizer prescription increased in the 20-59-year-old age group; also, these individuals comprised a high proportion (>90%) of internet users during the past several years [6,31]. If patients’ ages crossed two age groups (ie, they moved into another age group during the course of the study), we included them in the younger group. We showed data regarding diagnoses related only to prescription heparinoid when more than 5% of patients were diagnosed with a disease of skin and subcutaneous tissue based on the ICD-10.

We adjusted the prescription time series according to the insured population’s data and showed prescriptions per 100,000 members of the population. We then selected two time series for cross-correlation. In correlating the time series, we accounted for trend and seasonality in time series data to avoid mistaken correlations. To examine the association between internet searches and moisturizer prescription, we used the Box-Jenkins approach to fit an autoregressive integrated moving average (ARIMA) model [32]. We used a seasonal ARIMA model, as all-time series data showed seasonality that increased from fall to winter and decreased from spring to summer annually. We performed the Kwiatkowski-Phillips-Schmidt-Shin test to render the series stationary and estimated the model parameters and diagnosed acceptability using the Akaike Information Criterion. We then checked the adequacy of the model, regardless of autocorrelation between residuals, using the Ljung-Box test. We performed cross-correlation analysis for each residual to assess the relationship between the two time series.

Cross-correlation analysis was performed for the following: (1) internet search trends for ”
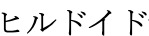
” (Hirudoid) and prescription trends (total) and (2) internet search trends for ”
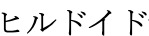
” (Hirudoid) and trends in heparinoid prescription in those aged 20-59 years. All trend data were calculated monthly. We used R, version 3.5.0 (The R Foundation), for statistical analysis.

## Results

**Figure 1:**
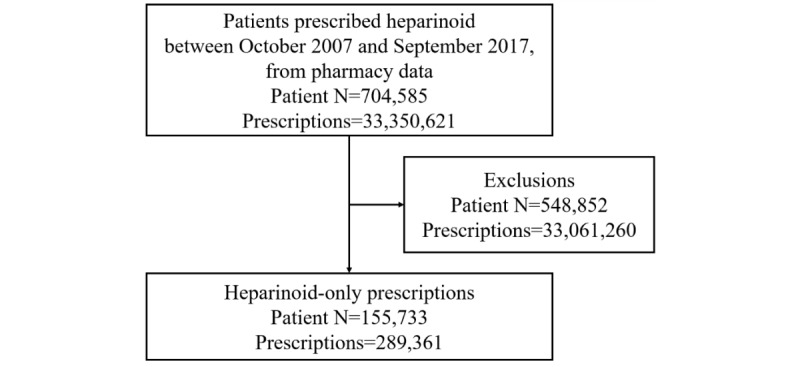
Study flowchart.

The pharmacy data showed that 704,585 patients (population A) were prescribed heparinoid along with other medicines between October 1, 2007, and September 31, 2017. In total, 155,733 patients (population B) were prescribed only heparinoid; of these, 70,819 (45.47%) were men and 84,914 (54.53%) were women (see Figure 1 and Table 1). The total number of prescriptions was 289,361 for all patients; 132,850 for men; and 156,511 for women. The most common diagnosis was xerosis cutis (ICD-10 code L853), followed by dermatitis, unspecified (ICD-10 code L309); atopic dermatitis, unspecified (ICD-10 code L209); and other atopic dermatitis (ICD-10 code L208). Compared to the characteristics of population A, the proportion of patients from population B with dermatitis, unspecified, and atopic dermatitis, unspecified, decreased by 50%.

**Table 1:** Patients’ baseline characteristics.

Characteristics			Population A: patients prescribed heparinoid along with other medicines			Population B: patients prescribed only heparinoid		
			Total (N=704,585)	Men (n=327,694)	Women (n=376,891)	Total (N=155,733)	Men (n=70,819)	Women (n=84,914)
**Age group (years), n (%)**								
	0-19		396,761 (56.31)	200,973 (61.33)	195,788 (51.95)	114,503 (73.53)	57,572 (81.29)	56,931 (67.05)
	20-59		278,625 (39.54)	111,891 (34.14)	166,734 (44.24)	36,421 (23.39)	11,194 (15.81)	25,227 (29.71)
	≥60		29,199 (4.14)	14,830 (4.53)	14,369 (3.81)	4809 (3.09)	2053 (2.90)	2756 (3.25)
**Diagnosis, n (%)**								
	Xerosis cutis		571,167 (81.06)	264,301 (80.65)	306,866 (81.42)	117,450 (75.42)	53,117 (75.00)	64,333 (75.76)
	Dermatitis, unspecified		301,528 (42.80)	138,321 (42.21)	163,207 (43.30)	34,234 (21.98)	15,539 (21.94)	18,695 (22.02)
	Atopic dermatitis, unspecified		196,975 (27.96)	101,037 (30.91)	95,938 (25.45)	22,656 (14.55)	11,191 (15.80)	11,465 (13.50)
	Other atopic dermatitis		60,584 (8.60)	31,581 (9.64)	29,003 (7.70)	11,769 (7.58)	6073 (8.58)	5696 (6.71)
**Prescriptions**								
	Total, N		3,350,692	1,680,423	1,670,269	289,361	132,850	156,511
	**Age group (years), n (%)**							
		0-19	2,182,796 (65.14)	1,150,733 (68.48)	1,032,063 (61.79)	226,044 (78.12)	113,415 (85.37)	112,629 (71.96)
		20-59	1,069,036 (31.90)	477,357 (28.41)	591,679 (35.42)	55,547 (19.20)	16,219 (12.21)	39,337 (25.13)
		≥60	98,860 (2.95)	52,333 (3.11)	46,527 (2.79)	7770 (2.69)	3225 (2.43)	4545 (2.90)

**Figure 2:**
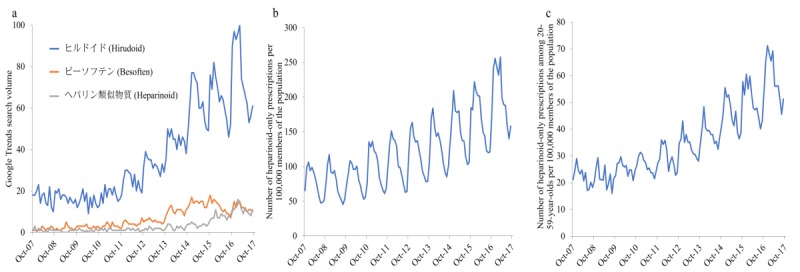
Time series data of Google Trends and prescriptions. (a) Google Trends search volume with three search terms from October 2007 to September 2017; search volume is the ratio of searches on each topic to searches on all topics, from 0 to 100. (b) Number of heparinoid-only prescriptions per 100,000 members of the population from October 2007 to September 2017. (c) Number of heparinoid-only prescriptions for patients aged 20-59 years per 100,000 members of the population from October 2007 to September 2017.

Heparinoid prescription increased from 2011 and peaked in the winter of 2017 (see Figure 2, b and c). Heparinoid prescription showed seasonality, in that it increased in the winter. Between October 1, 2007, and September 31, 2017, “
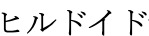
” (Hirudoid) was the most frequently searched term of the three terms entered into Google Trends (see Figure 2, a). In addition, “
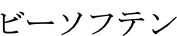
” (Besoften) and “
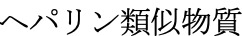
” (heparinoid) showed similar search volumes. The search volume for “
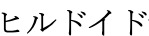
” (Hirudoid) increased from 2012 and peaked in the winter of 2017.

The trend data allowed us to define a seasonal ARIMA model for each time series. Google Trends data, trends in total prescriptions, and prescription trends in patients aged 20-59 years fit the seasonal ARIMA model (5,1,4 and 0,1,1; 2,1,5 and 2,1,0; and 4,1,5 and 0,1,1 (12), respectively) (see Table 2). Cross-correlation analysis of ARIMA residuals for two time series showed a positive correlation (see Table 3). Trends in total prescription peaked at the 0-month lag, with a correlation coefficient of .25 (*P*=.005). Prescription trends for patients aged 20-59 years peaked at the −1-month lag, with a correlation coefficient of .30 (*P*=.001).

**Table 2:** ARIMA^a^ model parameter and Q statistics^b^.

Time series	Model	AIC^c^	Q statistic	*P* value
Google Trends “ 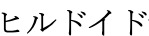 ” (Hirudoid)	Seasonal ARIMA (5,1,4 and 0,1,1) (12)	704	17.7	.22
Prescriptions (total)	Seasonal ARIMA (2,1,5 and 2,1,0) (12)	823	19.4	.19
Prescriptions (patients aged 20-59 years)	Seasonal ARIMA (4,1,5 and 0,1,1) (12)	567	23.3	.055

^a^ARIMA: autoregressive integrated moving average.

^b^Q statistics test for residual autocorrelation. The null hypothesis is that autocorrelation with 24 lags would be equal to zero.

^c^AIC: Akaike Information Criterion.

**Table 3:** Cross-correlation between prescriptions and Google Trends for “
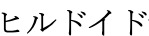
” (Hirudoid).

Prescriptions	Lag (months)	Correlation coefficient	*P* value
Total	0	.25	.005
Patients aged 20-59 years	–1	.30	.001

## Discussion

### Principal Findings

The results showed that heparinoid prescriptions increased from 2012 onward and that “
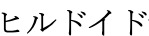
” (Hirudoid) was the most frequently searched of the three terms entered into Google Trends. In addition, prescription of heparinoid only had a weak positive correlation with internet search data obtained from Google Trends.

A previous study showed that drug utilization time series for antibiotics—amoxicillin, azithromycin, and cefdinir—and Google Trends were correlated [28]. This study showed similar results for heparinoid prescription. In particular, the results for the population aged 20-59 years correlated to a −1-month lag in Google Trends. This implies that there was a possibility that this population was affected by the internet and/or that they tend to seek out medical information before going a doctor. We cannot assess this relationship directly; however, we showed that there is a relationship between medical prescriptions and internet searches.

There are many reasons why people search for health-related information using the internet [33]. For instance, they may perform searches for a newly diagnosed health problem, ongoing medical condition, newly prescribed medication or treatment, or health-related knowledge. Therefore, people could search before or after undergoing medical treatment [34]. In this study, Google Trends showed that “
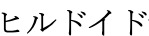
” (Hirudoid) was the most commonly searched term related to heparinoid in patients aged 20-59 years. This result suggested that people visited a physician after obtaining information regarding heparinoid; it also suggested that internet searching affected their behavior and physicians’ decision making.

Many people were prescribed heparinoid in a clinic or hospital in this study, even though heparinoid is sold in pharmacies as an over-the-counter (OTC) medicine. There could be several reasons for this. First, people have to obtain a prescription from a physician to get Hirudoid, specifically. While people can get OTC medicines containing heparinoid, they cannot buy Hirudoid without a prescription. Results of Google Trends showed that *Hirudoid* was the most frequently searched of the three terms; this could be the main reason that people sought prescriptions. Second, because of the way the Japanese health care system works, some Japanese people might choose to visit clinics or hospitals for prescribed medicine in order to obtain it at a cheaper rate relative to the OTC price. In Japan, everyone has some type of insurance because of the national health insurance system. Therefore, they pay up to 30% of the total medical expenditure in hospitals, clinics, and pharmacies [35]. In a past questionnaire survey, 36.1% of patients did not consider the use of OTC drugs before visiting medical institutions. In addition, 40.5% of participants answered that visiting medical institutions was cheaper [36]. When people choose whether to visit a medical institution or buy OTC drugs, there are many reasons that affect their decision [37]. However, this could explain why people visit clinics or hospitals rather than buying OTC medicines.

Patients’ medical decision making is changed though health information, regardless of credibility [7,18]. A previous study showed no difference in the effects of high- and no-credibility sources [38]; judgment of credible information could be difficult for many consumers. Health literacy has been considered an important factor affecting health behavior and health information access [39-41]. However, levels of health literacy among the Japanese population are lower relative to those observed among the European population. They concluded that there are few reliable, understandable Japanese websites such as MedlinePlus, as well as a lack of health communication specialists and an inefficient primary health care system in Japan [42]. Moreover, some patients have explained that they do not use OTC drugs because they do not know which OTC drugs are the highest quality or most suitable for their symptoms and they cannot be trusted [36]. This may imply that people do not have the opportunity to learn how to obtain the correct information. Thus, education to enable people to achieve high internet health literacy levels and the ability to search and judge credible information is more important than ever.

On October 31, 2018, the Japanese Dermatological Association reported on medical heparinoid prescriptions for cosmetic purposes and stated that members should prescribe it appropriately; if it is prescribed for cosmetic application, patients should pay for the medication themselves [43]. In addition, the Central Social Insurance Medical Council developed two policies on February 7, 2018. The first policy states that heparinoid prescriptions shall not be covered for the purpose of cosmetic use to promote circulation and skin moisturization (ie, heparin sodium and heparinoid) [44]. The second policy states that examination and payment agencies should deal with prescriptions appropriately. However, prescriptions were not limited because of a lack of evidence regarding heparinoid prescription. Therefore, these findings provide important evidence for trends in heparinoid prescription.

### Limitations

This study was subject to several limitations. For example, the JMDC database contains data for employees of large corporations and their family members. However, because of a lack of data for employees of small and medium-sized businesses, public officials, and self-employed people, the results cannot be generalized to the wider population in Japan. In addition, Google and Yahoo are the main internet search engines used in Japan [45], and Google Trends does not include the entire Japanese population. However, the use of Google is increasing and it may, therefore, represent most Japanese people. Moreover, Google Trends shows data only for search queries; we did not have access to details about how research words were recognized and aggregated on Google or the information obtained using specific search terms. This study examined only the association between internet searches and prescriptions. Therefore, it did not clarify the cause of the increase in prescriptions or the number of people prescribed the moisturizer for cosmetic purposes due to a lack of detailed information about attitudes and prescription behaviors. To elucidate this relationship, another approach is needed; for example, a questionnaire survey administered to physicians or patients could assess moisturizer prescriptions for cosmetic purposes.

### Conclusions

This was the first study to report on the association between internet searches and prescriptions in Japan. The results of the analysis suggested that internet searching for health-related information affected heparinoid prescription, although the positive correlation between these variables was weak. In particular, prescriptions for people aged 20-59 years correlated with the –1-month lag in Google Trends. Seeking internet-based information changed peoples’ behavior and physicians’ prescription habits. Also, Google Trends quantitatively demonstrated peoples’ interest and could be effective in detecting changes in drug utilization. The internet provides beneficial information to patients, medical personnel, and healthy people for decision making. However, it is not easy to distinguish between correct and incorrect information because of the volume included.

## Abbreviations

AIC: Akaike Information Criterion
ARIMA: autoregressive integrated moving average
ICD-10: International Statistical Classification of Diseases and Related Health Problems, Tenth Revision
OTC: over-the-counter
